# Comparative analysis of alternative splicing events in skeletal muscle of different sheep

**DOI:** 10.1016/j.heliyon.2023.e22118

**Published:** 2023-11-08

**Authors:** Xiangyang Miao, Qingmiao Luo, Huijing Zhao, Xiaoyu Qin

**Affiliations:** Institute of Animal Sciences, Chinese Academy of Agricultural Sciences, Beijing, 100193, China

**Keywords:** Sheep, Muscle, Alternative splicing, RNA-seq, mRNAs

## Abstract

This paper aims to investigate the relationship between genes with alternative splicing (AS) events and breed-specific differences in muscle development in two breeds of sheep. RNA-seq was utilized to identify genes with AS between Small-tailed Han sheep and Dorset sheep. The gene lists of differentially spliced genes were identified, and Gene Ontology (GO) and Kyoto Encyclopedia of Genes and Genomes (KEGG) pathway enrichment analysis were conducted on these genes. In this study, 299 genes with 356 AS indicated significant differences between two diffrerent breeds. There are differences in 31 genes with 35 AS. Cassette, alt5′ and alt3’ exhibited the highest levels of enrichment across various significant levels. GO and KEGG enrichment analysis demonstrated a significant correlation between Wnt, TGF-beta, Notch and MAPK signaling pathways and the development of muscle in sheep. These findings indicate that genes with AS are linked to variations in muscle development in sheep. These results offer significant scientific and practical implications for improving the quality of sheep meat.

## 1 Introduction

Sheep (*Ovis aries*) are common domestic animals, utilized as a source of meat, milk, and other products. The Small-tailed Han sheep, a sheep breed native to the Yellow River region of China, is an important commercial breed with excellent fertility and medium-to-large size [1]. The genetics of Small-tailed Han breed have been well studied [[2], [3], [4]]. Several genetic studies of this breed have focused on identifying gene signatures linked with desirable traits, such as high fertility [[5], [6], [7], [8]]. Small-tailed Han sheep (Han sheep) have higher fat content, tasting meat and a slower growth rate. However, Dorset sheep are a breed hailing from England with desirable muscle structures and meat production. It has a rapid growth rate with a large and lean carcass that make them ideal for the meat trade. Significant differences in fat levels and utilizations between the two different sheep have raised interest in potential genetic factors regulating fat deposition. This can greatly improve meat quality. Determining the molecular mechanisms of fat deposition can lead to better control of the quality and nutritional content of sheep meat. Therefore, understanding the genetic profiles of the muscle can promote sheep growth and meat production.

Many studies compared sheep breeds based on next generation sequencing, including the comparison of transcriptomes in Surabaya fur sheep with Han sheep [9], and the comparison of muscles and mRNAs in Han and Dorset sheep [[10], [11], [12], [13]]. Alternative splicing is a molecular process whereby the different exons present within a given gene's primary mRNA transcript (pre-mRNA) are spliced together in different conformations such that upon translation, the resultant proteins are functionally and conformationally distinct [14]. This process is a common method that can increase the complexity and diversity of eukaryotic cells and organisms without additional genes. Therefore, protein isoforms can be produced that differ in protein-protein interaction, subcellular localization, or catalytic capacity [15]. Many studies about the alternative splicing of specific transcripts of interest, genome-wide sequencing studies indicate that AS can be utilized to alter the regulation of upwards of 95 % of genes in human cells encoded on more than one exon, with over 100,000 distinct AS events detected in one study [16]. Additionally, researches of mammalian gene expression indicated the wide spread of alternative transcription initiation and termination. This is the origin of the transcriptomic diversity [17]. There are various types of alternative events (AEs) between different parts of transcripts. They have different prevalences and functional significances. The utilization of AS across cell types can promote transcriptomic diversity. AEs that occur in a given transcript are not random. In fact, AS occurs most frequently in 5′ untranslated regions (5′UTRs) and coding sequences (CDSs), and 5′ UTRs is the most frequently affected sites of AS [18]. In contrast, AS of 3′UTRs is very rare because of few genes contain introns in this region [19]. Therefore, eukaryotic transcription is an extremely complex process that combines such AEs with differential capping and other regulatory activities to markedly alter gene expression within cells [20]. Interestingly, these results indicate that AEs affect expression and transcriptional regulation. In this study, we assessed genes that undergo differential AS in two species of sheep as a means of identifying the pathways linked with the regulation of muscle development. We hypothesized that there are differences in genes with AS events. Cassette, alt5′ and alt3′ were the most enriched items on various significant levels. GO and KEGG enrichment analysis indicated that Wnt, TGF-beta, Notch and MAPK signaling pathways were closely associated with muscle development. This paper aims to study genes with alternative splicing events in two breeds of sheep to investigate the relation between these events and breed specific differences in muscle development.

## 2 Materials and methods

### 2.1 Sample preparation

Two year-old Han and Dorset ewes were from the Qingdao Aote Sheep Farm (Shandong, China). Both groups of sheep had free access to food and water, and were housed in natural lighting before sacrifice. We collected longissimus dorsi muscle samples from two breeds of sheep. Animals have a period of fasting from solid food before sacrifice. Those animals were raised free range. Samples were snap-frozen in liquid nitrogen, and stored at −80 °C. Five sheep per breed were used for this study. The weight and carcass weight of the selected sheep were close to the median of the corresponding breed. The body weight and carcass weight were 75.3 kg, 33.8 kg for the Han sheep, and 80.5 kg, 37.8 kg for the Dorset sheep, respectively.

### 2.2 Experimental design

#### 2.2.1 mRNA library construction and sequencing

The experiment was performed in the Institute of Animal Sciences, Chinese Academy of Agricultural Sciences, Beijing, China. We used TRIzol (Invitrogen, Carlsbad, CA, USA) following instructions to isolate RNA from muscle samples. DNA was removed via RNase-free DNase I (Ambion, Austin, TX, USA). Then, Oligo (dT) magnetic beads were used to enrich mRNA, which was fractionated into ∼200bp fragments. The first strand cDNA was then produced via reverse transcription, while the second strand was produced with DNA polymerase. The resultant cDNA was then amplified to yield the mRNA library for sequencing. We performed RNA-sequencing via the Illumina genome analyzer IIX (the Shanghai Biotechnology Corporation, Shanghai, China). We confirmed the size and purity of the library via the Agilent 2100 system with a high sensitivity DNA chip. A Qubit™ dsDNA HS kit and Qubit® 2.0 fluorometer were utilized for quality control. The cBot of Illumina Genome Analyzer IIX was used for cluster generation.

#### 2.2.2 Alternative splicing analysis

After removing any unmapped or low-quality reads, uniquely mapped reads were aligned to a sheep reference genome (release Oar_4.0-sheep reference genome) using TopHat v2.0.6. Fragments per kilobase of transcript per million mapped reads (FPKM) were used to accommodate sequencing data with reads from a single source of molecules. We reconstructed and merged transcriptomes for each sample to generate an annotated transcript set for AS analyses. We utilized the AS detector program to detect possible AS events based on the following workflow: (i) exon clusters were reconstructed to identify common modes of AS for each cluster. (ii) Junction reads aligning with sites were considered as inclusion or exclusion sites for particular isoforms were then counted, and a *P*-value was calculated accordingly. (iii) We calculated read coverage for all alternatively spliced exons and the corresponding gene across samples. Another *P*-value was calculated by Fisher's exact test. (iv) These two *P*-values were combined to produce the adjusted *P*-value for assessing significant differences in AS between these sheep. AS levels were assessed based on the relative number of events per species, and those with similar expression levels between species were used to compare of AS rates. Differences in AS were significant if it was equal or greater than 4 times in a given breed relative to the other, or if it was present in only one of the two breeds.

#### 2.2.3 GO and KEGG analyses

Those genes with differential AS events between breeds were converted to ENSG symbols with gprofile (http://biit.cs.ut.ee/gprofiler/gconvert.cgi). We conducted KEGG analysis to gain insights into the general functional pathways and systems shared by differentially spliced genes and multiple levels of activities (http://www.genome.jp/kegg/). We assessed the enrichment of differentially spliced genes for those associated with particular KEGG pathways via KOBAS software [21,22]. The blastn was used to map mouse mRNAs via the default settings for Gene Ontology [23] annotation. The Mouse-Genome Informatics (MGI) annotations were assigned to corresponding sheep homologs of annotated mouse genes. The resultant list was then analyzed with GOEAST (http://omicslab.genetics.ac.cn/GOEAST/)) [24]. *P*-values and FDRs were calculated via Fisher's exact test.

### 2.3 Statistical analysis

All data are showed as the mean ± SD. The significant differences between group data was determined by the analysis of variance followed by the student's t-test for variance equality of using SPSS 17.0 (IBM, USA). P < 0.05 was considered statistically significant.

## 3 Results

### 3.1 Sequencing

A total of 79,804,368 and 75,271,338 clean reads were obtained from Dorset and Han sheep, respectively. And a total of 66,992,171 and 70,975,703 reads were mapped to Han and Dorset sheep, respectively. The analysis of AS was based on the mapped reads.

### 3.2 AS analysis of the sheep transcriptome

**Fig. 1:**
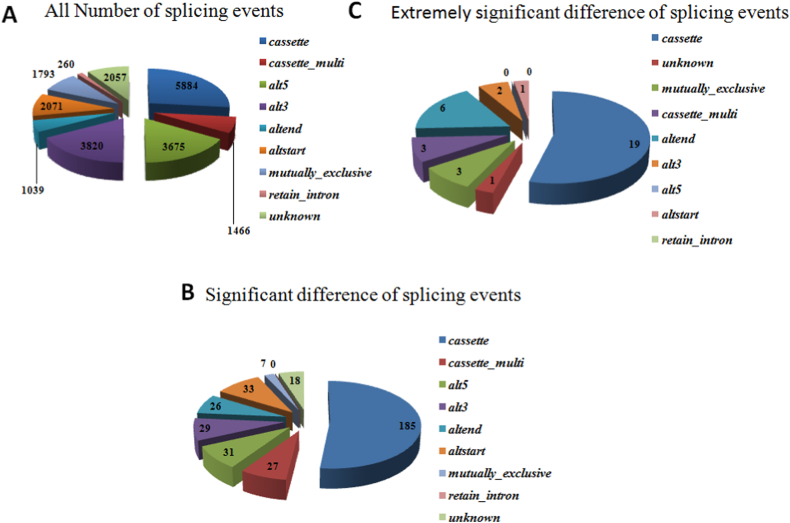
The alternative splicing events identified from two sheep breed. A: all alternative splicing events identified in this study. B: the identified alternative splicing events with significant difference. C: the identified alternative splicing events with extremely significant difference.

A total of 21,577,160 and 23,409,716 junctions were from Han and Dorset sheep, respectively. All reads were then identified that mapped to these identified splicing junctions, with nine different possible splicing junction types as follows: cassette, cassette_multi, alternative 3′ splicing site, alternative 5′ splicing site, altend, altstart, mutually_exclusive, retain_intron and unknown were considered in this study. The detailed distributions of AS events are shown in Fig. 1A. A total of 22,065 AS events had been identified between two breeds, and these events were located in 7985 genes. In Table S1, cassette (26.7 %; 5884 of 22,065), alt5’ (16.7 %; 3675 of 22,065) and alt3’ (17.3 %; 3, 820 of 22,065) were the most enriched items, while retain_intron (1.2 %; 260 of 22,065) were the rarest items. The distributions of AS with significant and extremely significant differences between these two sheep were shown in Fig. 1B and C. Among AS events with significant differences, 297 genes with 356 AS events were detected. In addition, cassette (52.0 %; 185 of 356) was the most enriched item, and none of items were assigned to retain_intron (Fig. 1B). 31 genes with 35 AS were obtained from AS with extremely significant differences. In addition, cassette (54.3 %; 19 of 35) was the most enriched item, and none of items were assigned to retain_intron and alt5’ (Fig. 1C). The results indicated that there is one or more AS events for one gene. Additionally, cassette was the most enrichment item (over 50 %) among these significantly and extremely significantly different genes.

### 3.3 Genes with AS between Han and Dorset sheep

**Fig. 2:**
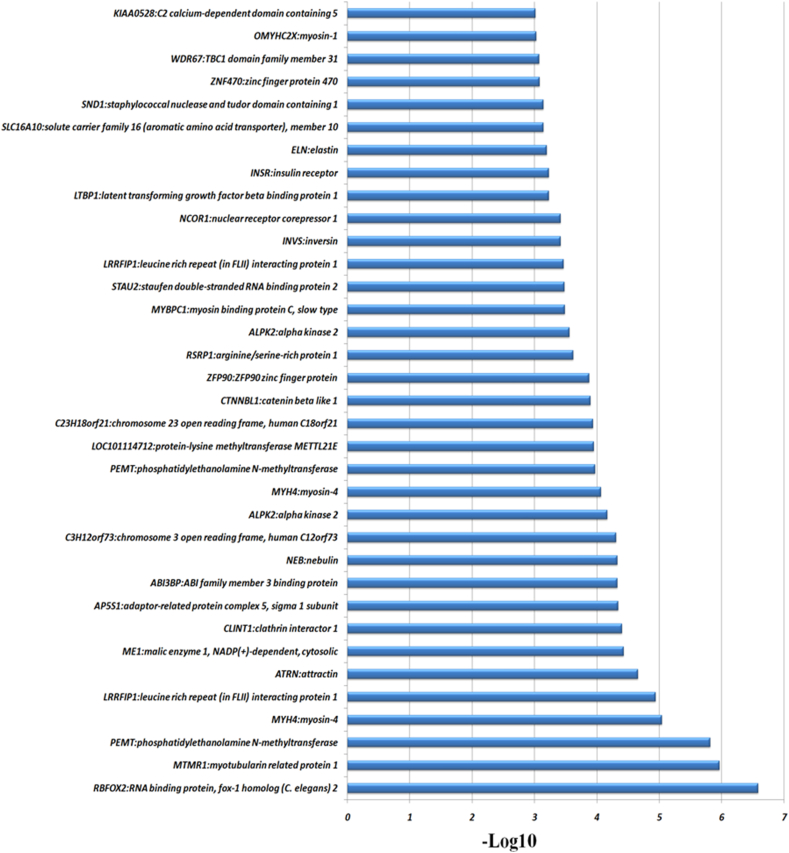
KEGG enrichment analysis of 245 genes with differential AS events identified. The significance level of enrichment was at –Log 10 (p value) > 0.5.

In total, 356 AS events were identified with differential frequencies between the two breeds of sheep. For example, RBFOX2, MTMR1, ME1, CLINT1, AP5S1 and ABI3BP were more common in the Han sheep than in Dorset sheep. Of the genes occurred AS with extremely significant differences, genes affected by cassette were RBFOX2, MTMR1, ME1, CLINT1, AP5S1, ABI3BP, C23H18orf21, RSRP1, ALPK2, MYBPC1, STAU2, INVS, NCOR1, LTBP1, INSR, ELN, ZNF470, OMYHC2X and KIAA0528. Unknown affected genes was PEMT. Alt3’ affected genes were C3H12orf73 and SND1. Altend affected genes were ATRN, ALPK2, PEMT, LOC101114712, ZFP90 and SLC16A10. Altstart affected genes was CTNNBL1. Cassette_multi affected genes were LRRFIP1, LRRFIP1 and WDR67. Mutually_exclusive affected genes were MYH4, NEB and MYH4 (Fig. 2). In Fig. 2, RBFOX2, MTMR1 and PEMT were the most enriched genes with extremely significant difference between Han sheep and Dorset sheep. RBFOX2 and MTMR1 were only affected by the cassette rather than other AS events.

### 3.4 KEGG analysis of differentially spliced genes

**Fig. 3:**
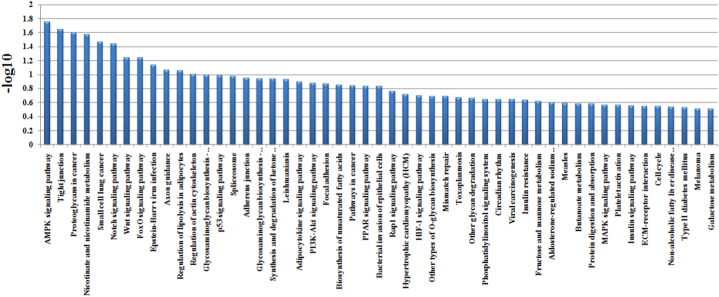
35 genes with extremely significant difference of the identified alternative splicing events. The significance level of enrichment was at –Log 10 (p value) > 3.

**Fig. 4:**
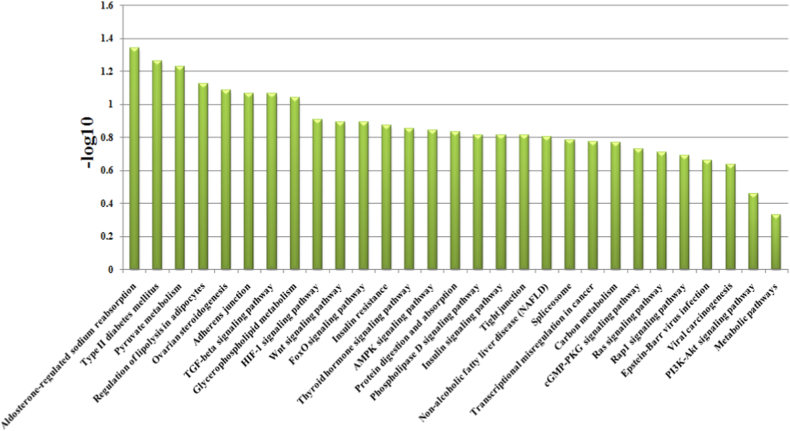
KEGG enrichment analysis of 27 genes with extremely significant difference AS events.

In this study, 297 genes with significant difference AS were involved in 111 pathways (Table S2). In Fig. 3, the pathway enrichment analysis indicated that AMPK signaling pathway was the top enriched term. Four genes were identified with significant difference AS that associated with this pathway. Tight junction, Proteoglycans in cancer, Nicotinate and nicotinamide metabolism, Small cell lung cancer and Notch signaling pathway were also significantly enriched. In addition, Wnt signaling pathway (oas04310; P value, 0.056), TGF-beta signaling pathway (oas04350; P value, 0.43), Notch signaling pathway (oas04330; P value, 0.036), MAPK signaling pathway (oas04010; P value, 0.27) were studied in this paper. In Table S3 and 31 genes with extremely significant difference AS were involved in 29 pathways. In Fig. 4, this pathway enrichment analysis indicate that Aldosterone-regulated sodium reabsorption was the top enriched term. A single gene was identified with significant difference AS in this pathway. Type II diabetes mellitus, Pyruvate metabolism, Regulation of lipolysis in adipocytes, Ovarian steroidogenesis, Adherens junction and TGF-beta signaling pathway were significantly enriched in the dataset. In addition, Wnt signaling pathway (oas04310; P value, 0.128), TGF-beta signaling pathway (oas04350; P value, 0.087) were present in this enrichment.

### 3.5 GO analysis of differentially spliced genes

**Fig. 5:**
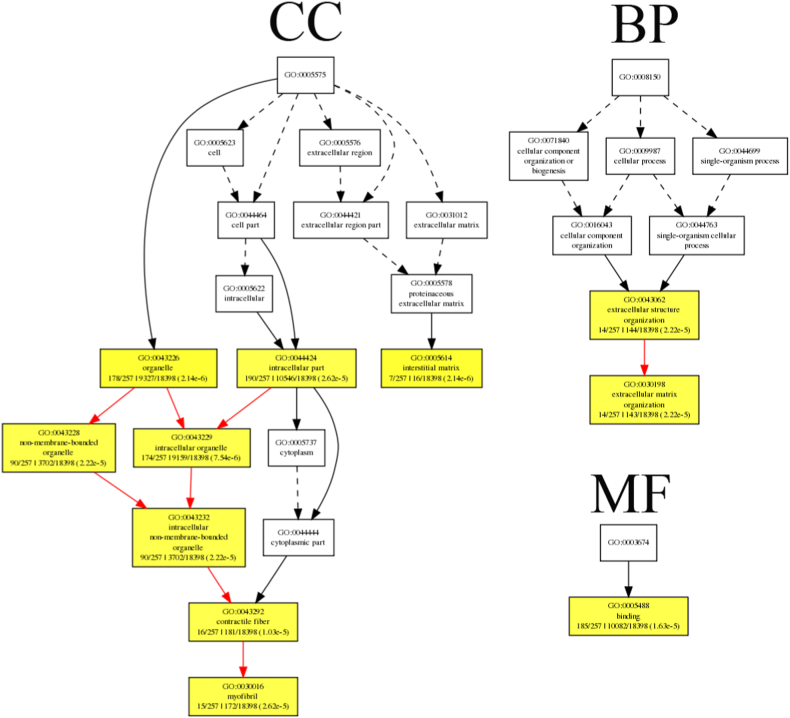
GO enrichment analysis of 297 genes with differential AS events. There are three parts: “biological processes (BP)”, “molecular functions (MF)”, and “cellular components (CC)”. In hypergeometric statistical test, the significance level of enrichment was set at p value <1e-4. Black solid lines symbolize the connections between enriched terms. The boxes contain GO functional positioning that is equivalent to the significant GO terms.

**Fig. 6:**
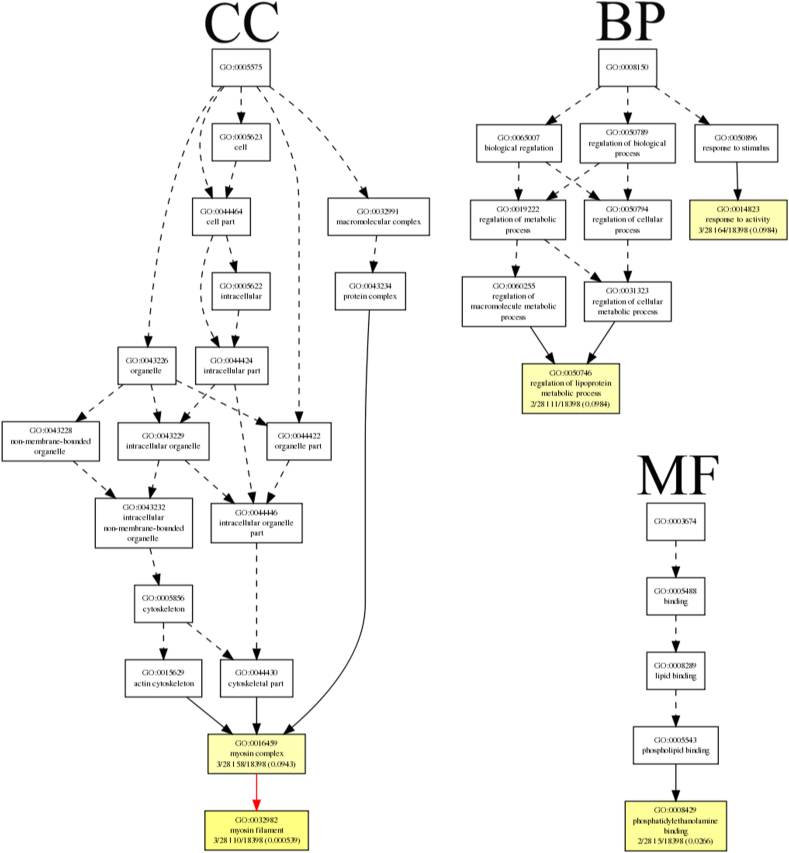
GO enrichment analysis of 31 genes with extremely differential AS events. There are three parts: “biological processes (BP)”, “molecular functions (MF)”, and “cellular components (CC)”. In hypergeometric statistical test, the significance level of enrichment was at p-value <0.1. Black solid lines represent the connections between enriched terms. The boxes contain GO functional positioning that is equivalent to the significant GO terms.

After screening, 297 genes with 356 significant differences AS were obtained. These 297 genes were appropriately bias-corrected for gene length using GOEAST (http://omicslab.genetics.ac.cn/GOEAST/index.php) [24]. In Fig. 5, terms with a corrected p < 1e-4 were significantly enriched. The functional roles of these different enriched genes could be divided into categories such as binding, organelle, intracellular organelle, intracellular part, non-membrane-bounded organelle, etc. GO terms were identified that are associated with categories of biological processes, cellular components, and molecular functions, as shown in Fig. 5 and Table S4. Two GO terms related to biological processes were assigned: extracellular matrix organization (GO: 0030198, P value: 2.22E-05) and extracellular structure organization (GO: 0043062, P value: 2.22E-05) were enriched. Eight GO terms under the cellular component were assigned. Among these terms, intracellular organelle (GO: 0043229, P value: 7.54E-06) was the top enriched term. One GO term was assigned in the catalogue of molecular function. Binding (GO: 0005488, P value: 1.63E-05) was the only significantly over-expressed terms. In addition, GO annotation and enrichment of all 31 genes with extremely significant difference were implemented. In this case, terms with p < 0.1 were significantly enriched to harvest as many terms as possible (Fig. 6). The identified gene functions can be classified into phosphatidylethanolamine binding, regulation of lipoprotein metabolic process, response to activity, myosin complex and myosin filament (Table S5). The different enriched GO terms are shown in Fig. 6 and Table S2. Two GO terms were assigned in the catalogue of biological processes. Among these terms, regulation of lipoprotein metabolic process (GO: 0050746, P value: 0.098) and response to activity (GO: 0014823, P value: 0.098) were enriched. Two GO terms were assigned in the catalogue of cellular components. Among these terms, myosin complex (GO: 0016459, P value: 0.094) and myosin filament (GO: 0014823, P value: 0.0005) were enriched. One GO term was assigned in the catalogue of molecular functions. The phosphatidylethanolamine binding (GO: 0008429, P value: 0.027) was the only significantly over-expressed terms.

## 4 Discussion

In this study, the sheep transcriptome was screened for differential AS event through a deep sequencing-based approach, the sequencing-by-synthesis (SBS) technique offered by Illumina platforms. This has been proven to be a highly accurate means of sequencing [[10], [11], [12], [13],[25], [26], [27], [28]]. We selected the Han and Dorset sheep in this study because of their significantly different growth rates and fat content. The differential muscle growth rates of these specific species affect the flavor and amount of meat yielded per animal. This is related to differential gene regulation that affects muscle growth rates. RNA sequencing is a valuable technique that is good for broad assessments of gene expression. It is more powerful than traditional data microarray-based approaches [14]. Many studies have used microarrays to assess the regulation of sheep muscle development, but few studies have identified differential AS event rates in sheep muscles via an RNA-seq-based approach. Therefore, we conducted analysis in the muscles of Han and Dorset sheep through an RNA-seq-based approach, and detected AS rates of 31.33 % (7895 of 25,197) for the reference genes with 22,065 AS events. This proportion was significantly less than the 86.0 % in humans [29], closer to the 33.0 % in rice [30], and greater than the 18.0 % in pigs [31]. In addition, we determined that cassette-type AS events were the most common type in sheep, unlike in rice where intron retention is the most common AS [30]. This was significantly different from results in humans and yeast, where exon-skipping is the most common AS event. In the future, it is necessary to study the differential regulation of AS events between breeds of sheep.

Compared with Han sheep, there were a total of 297 differentially spliced genes in Dorset sheep, including a total of 356 different splicing differences. The KEGG analyses of these 297 genes, and separate analysis of 31 significantly differentially spliced genes showed that the Wnt, Notch, MAPK, and TGF-beta signaling pathways were all enriched among these genes. This indicates that they are involved in muscle development in these sheep. This is consistent with the results of previous researches. This indicates that Wnt signaling can drive fibrosis in muscles with pro-fibrotic canonical Wnt/β-catenin signaling altering ECM makeup [32]. The Wnt pathway can further interact with other pro-fibrotic events, such as connective tissue growth factor (CTGF) and TGF-β signaling which have been shown to drive fibrosis in organs including liver, kidney and skeletal muscle [33]. Notch signaling is a conserved pathway essential for tissue development and the maintenance of normal tissue homeostasis [34]. Notch signaling in skeletal muscle is essential for determining the fate and proliferative capacity of muscle cells during tissue regeneration. The exact spatial and temporal dynamics of Notch signaling affect the way that regenerated tissue forms. Governing and controlling Notch signaling with a complex series of regulatory pathways can promote the proper localization, stabilization, and activation of necessary signaling proteins [35]. Multiple different secreted growth factors affect the development of skeletal muscle, altering transcriptional programs within the cell. The p38 mitogen-activated protein kinase (MAPK) pathway is important for the signal transduction downstream of such growth factors. Altering p38 signaling in muscle cells can significantly alter expression patterns of genes related to muscle function. Altering transcription factors is important for muscle cells such as MyoD and MEF2, and shapin chromatin remodeling in areas of the genome encoding muscle proteins. p38 signaling can cooperate with other transcription factors in muscle cells to altering the regulation of certain genes later in muscle differentiation, thereby altering the temporal dynamics of the differentiation. These results have been validated in recent studies of myogenesis in Xenopus embryos [36]. GO analysis of these genes with significant difference AS shows that they can be categorized into various processes, including binding, organelle, intracellular organelle, intracellular part, non-membrane-bounded organelle, intracellular non-membrane-bounded organelle, myofibril, contractile fiber, interstitial matrix, extracellular matrix organization and extracellular structure organization. This is consistent with the development of muscle. For example, myofibril and contractile fiber are closely related with the formation and differentiation of muscle cells. Furthermore, there were 31 genes with extremely significant differences between two breeds. These genes were linked with the stress and unfolded protein responses, myoblast cell fate determination, and the extracellular matrix. For example, Rbfox2 with the most significant difference AS events had been proven the closely relationship with muscles development. In *C. elegans*, members of the Rbfox homolog family are known to mediate splicing events, which are specific to muscle cells [37]. This has also been found in zebrafish, in which such Rbfox homologs affect muscle development in heart and skeletal muscle tissues. Therefore, knocking down these proteins can lead to abnormal development, reduce heart rates, and muscle paralysis. Although Rbfox1 and Rbfox2 are known to be expressed at high levels in human skeletal muscle tissue [38]. Many Rbfox splicing targets have not been identified. Myotubularin-related 1 gene (MTMR1) is also associated with altered differentiation of mus*cle cells that is* associated with congenital myotonic dystrophy type 1 (DM1) [39]. MTMR1 expression is carefully regulated during the differentiation of muscle cells. Appropriate AS events cause adult cells to accumulate higher levels of the adult-specific C isoform of MTMR1 and lower levels of the fetal A and B isoforms [39]. Heat shock proteins (HSPs) are important stress response proteins with multiple roles, including acting as chaperones to promote correct protein folding. Disrupting HSP activity can lead to the induction of unfolded protein responses. Therefore, differentially spliced HSP proteins in the muscles of Han and Dorset sheep can affect muscle's response to stress, thereby affecting the quality of the meat. It is necessary to study the impact of these genes and splicing events on the progression of muscle development in future.

## 5 Conclusions

This study investigated the relationship between genes with alternative splicing (AS) events and breed-specific differences in muscle development in two breeds of sheep. 299 genes with 356 AS events exhibited significant differences between the two breeds, including 31 genes with 35 AS events. Cassettes, alt5′, and alt3′ were the most commonly enriched items. Additionally, GO and KEGG enrichment analyses suggested that the Wnt, TGF-beta, Notch, and MAPK signaling pathways were closely associated with muscle development. These findings demonstrate that genes with AS events are linked to differential muscle development in sheep, and offer valuable scientific and practical insights for improving sheep meat quality in the future. This indicates that AS events with significant differences can affect those pathways. This study provides key insights that prove to be valuable for novel efforts to select sheep with superior meat quality.

## Data availability statement

Data included in article/supp. material/referenced in article.

## Ethics statement

All the procedures involving animals were approved by the animal care and use committee at the Institute of Animal Sciences, Chinese Academy of Agricultural Sciences (NO. IAS2019-82), where the study was performed. All the experiments were conducted in accordance with the relevant guidelines and regulations set by the Ministry of Agriculture of the People's Republic of China.

## Funding statement

Professor Xiangyang Miao was supported by the Major Science and Technology Project of New Variety Breeding of Genetically Modified Organisms (Nos. 2009ZX08008-004), the Agricultural Science and Technology Innovation Programme (ASTIPIAS05) and the Basic Research Fund for Central Public Research Institutes of 10.13039/501100005196CAAS (Y2016JC22, Y2018PT68).

## CRediT authorship contribution statement

**Xiangyang Miao:** Writing – original draft, Visualization, Supervision, Project administration, Funding acquisition, Conceptualization. **Qingmiao Luo:** Validation, Software, Resources, Methodology, Investigation, Formal analysis, Data curation. **Huijing Zhao:** Software, Resources, Investigation, Formal analysis, Data curation. **Xiaoyu Qin:** Validation, Software, Resources, Methodology, Investigation, Formal analysis, Data curation.

## Declaration of competing interest

The authors declare no conflict of interest.
